# NLRC5 knockdown in chicken macrophages alters response to LPS and poly (I:C) stimulation

**DOI:** 10.1186/1746-6148-8-23

**Published:** 2012-03-08

**Authors:** Ling Lian, Ceren Ciraci, Guobin Chang, Jingdong Hu, Susan J Lamont

**Affiliations:** 1 Department of Animal Science, Iowa State University, Ames, IA 50011, USA; 2 Department of Animal Genetics and Breeding, National Engineering Laboratory for Animal Breeding, MOA Laboratory of Animal Genetics and Breeding, College of Animal Science and Technology, China Agricultural University, Beijing 100193, China; 3 College of Animal Science and Technology, Yangzhou University, Yangzhou, Jiangsu 225009, China; 4 College of Veterinary Medicine, Shandong Agricultural University, Taian, Shandong 271018, China

## Abstract

**Background:**

NLRC5 is a member of the CARD domain containing, nucleotide-binding oligomerization (NOD)-like receptor (NLR) family, which recognizes pathogen-associated molecular patterns (PAMPs) and initiates an innate immune response leading to inflammation and/or cell death. However, the specific role of *NLRC5 *as a modulator of the inflammatory immune response remains controversial. It has been reported to be a mediator of type I IFNs, NF-kB, and *MHC class I *gene. But no study on *NLRC5 *function has been reported to date in chickens. In the current study, we investigated the role of *NLRC5 *in the regulation of *IFNA*, *IFNB*, *IL-6*, and *MHC class I *in the chicken HD11 macrophage cell line, by using RNAi technology. HD11 cells were transfected with one of five siRNAs (s1, s2, s3, negative-siRNA, or a mixture of s1, s2, s3-siRNAs). After 24 hours, cells were exposed to LPS or poly (I:C) or a vehicle control. Gene expression of *NLRC5*, *IFNA*, *IFNB*, *IL-6*, and *MHC class I *at 2, 4, 6, and 8 hours post stimulation (hps) was quantified by qPCR.

**Results:**

The expression of *NLRC5*, *IFNA*, *IFNB*, and *IL-6 *genes in negative irrelevant transfection controls was up-regulated at 2 hps after LPS treatment compared to the vehicle controls. S3-siRNA effectively knocked down *NLRC5 *expression at 4 hps, and the expression of *IFNA *and *IFNB *(but not *IL-6 *and *MHC class I*) was also down-regulated at 4 hps in s3-siRNA transfected cells, compared to negative irrelevant transfection controls. Stimulation by LPS appeared to relatively restore the decrease in *NLRC5*, *IFNA*, and *IFNB *expression, but the difference is not significant.

**Conclusions:**

Functional characterization of chicken *NLRC5 *in an *in vitro *system demonstrated its importance in regulating intracellular molecules involved in inflammatory response. The knockdown of *NLRC5 *expression negatively mediates gene expression of *IFNA *and *IFNB *in the chicken HD11 cell line; therefore, *NLRC5 *likely has a role in positive regulation of *IFNA *and *IFNB *expression. No direct relationship was found between *NLRC5 *knockdown and *IL-6 *and *MHC class I *expression. Future studies will further clarify the roles of *NLRC5 *and other NLRs in infectious diseases of chickens and may increase the efficacy of antiviral vaccine design.

## Background

The host innate immune system recognizes various pathogen-associated molecular patterns (PAMPs) and danger-associated molecular patterns (DAMPs) through pattern-recognition receptors (PRRs) and triggers the inflammatory response to defense against microorganisms invasion [1]. There are three classes of PRRs in vertebrates, Toll-like receptors (TLRs), nucleotide-binding oligomerization (NOD)-like receptors (NLRs), and the retinoid acid-inducible gene-I (RIG-I)-like receptors (RLRs) [1-6]. These molecules showed different subcellular localization, for example, most TLRs identify extracellular PAMPs, whereas NLRs and RLRs sense intracellular PAMPs [7,8]. Recently, the NLRs have gained attention because of their involvement in mediating innate immune responses to microbial invasion and controlling innate immune pathways. To date, at least 22 members of the NLR family have been identified in humans [9], and some of them have been well characterized. For example, two members of the NLRC (NLR containing a caspase-recruitment domain (CARD)) family, NLRC1 and NLRC2, recognize bacteria-derived molecules and result in activation of downstream signaling pathways, including NF-kB and mitogen-activated protein kinase (MAPK)s, to induce production of inflammatory cytokines [10,11]. NLRP1 and NLRP3 (NLR containing a pyrin domain) play key roles in activating caspase-1 inflammasomes as a response to PAMPs and DAMPs, leading to maturation and secretion of pro-inflammatory cytokines interleukin (IL)-1B and IL-18 [12]. In addition, *NLRC1*, *NLRC2*, *NLRP1*, *NLRP3*, and *MHC class II *transactivator (*CIITA*) are all associated with susceptibility to chronic inflammatory diseases [13-20].

Recently, NLRC5, a newly identified member of NLR family, with the most evolutionary relationship to NLRC1 and NLRC2 [21,22], has been implicated in regulation of the innate immune response [23]. *NLRC5 *is highly expressed in lymphocytic and macrophage/monocytic cell lineages [24] and immune tissues [9]. *NLRC5 *can be potently induced by interferon gamma (IFNG) and modestly induced by the microbial-derived molecules, lipopolysaccharide (LPS) and polyinosinic:polycytidylic acid (poly (I:C)) [9]. The specific role of *NLRC5 *in acting as a regulator of proinflammatory pathways is controversial [24]. *NLRC5 *is suggested to positively regulate the interferon (IFN) pathway in human cervical carcinoma HeLaS3 cells and human acute monocytic cell line, THP-1 [25]. Overexpression of *NLRC5 *leads to the activation of IFN-specific response elements (ISRE) in HeLa cells [25]. siRNA-mediated knockdown of endogenous *NLRC5 *decreased type I IFN pathway-dependent responses mediated by Sendai virus and poly (I:C) in THP-1 cells [26]. In contrast, overexpression of *NLRC5 *was shown to repress the activation of NF-kB-, type I IFN-, and AP-1-dependent signaling pathways in human embryonic kidney cell line HEK293T, and the knockdown of *NLRC5 *increased secretion of proinflammatory cytokines in mouse leukaemic monocyte macrophage cell line, RAW264.7 [9]. Moreover, Cui et al. demonstrated that *NLRC5 *negatively modulated NF-kB and type I IFN signaling pathways and its absence resulted in elevated expression levels of *Tumor necrosis factor *(*TNF*) *A*, *IL-6*, and *IL-1B *[27]. In addition, opposite effects have also been described for the role of *NLRC5 *as a transcriptional regulator of *MHC class I *expression. It binds to the *MHC class I *promoter and up-regulates *MHC class I *expression in lymphoid and epithelial cell lines. *NLRC5 *was also proven to be required in the pathway of efficient induction of *MHC class I *by IFNG stimulation [28]. In contrast, knockdown of *NLRC5 *was shown to increase cell surface expression of MHC class I in RAW 264.7 cells, which indicated a negative role of *NLRC5 *in regulating *MHC class I *expression [9]. Therefore, the effects of *NLRC5 *in regulating immune-related components and inflammatory responsive pathways are very complex, and likely either cell type- or species-specific [24].

To date, no study on the functional role of *NLRC5 *in chickens has been reported. In the current study, we characterized the functions of *NLRC5 *gene in the regulation of *IFNA, IFNB, IL-6*, and *MHC class I *by utilizing RNA-interference technology and stimulation with LPS from *Salmonella typhimurium *or poly (I:C) in the chicken HD11 macrophage cell line. These specific ligands were selected to complement and expand upon our previously published studies [29-31]. The expression levels, over time, for five genes were determined by qPCR to elucidate the association of *NLRC5 *with *IFNA*, *IFNB*, *IL-6*, and *MHC class I. *The present study reports the initial characterization of chicken *NLRC5 *and its roles in the regulation of innate immune response to bacterial components. Our findings are novel and of significant scientific value for a better understanding of host response to infectious diseases in chickens.

## Results and discussion

### Expression of *NLRC5, IFNA, IFNB*, and *IL-6 *in negative irrelevant siRNA transfection controls was up-regulated at 2 hours post LPS stimulation

**Figure 1:**
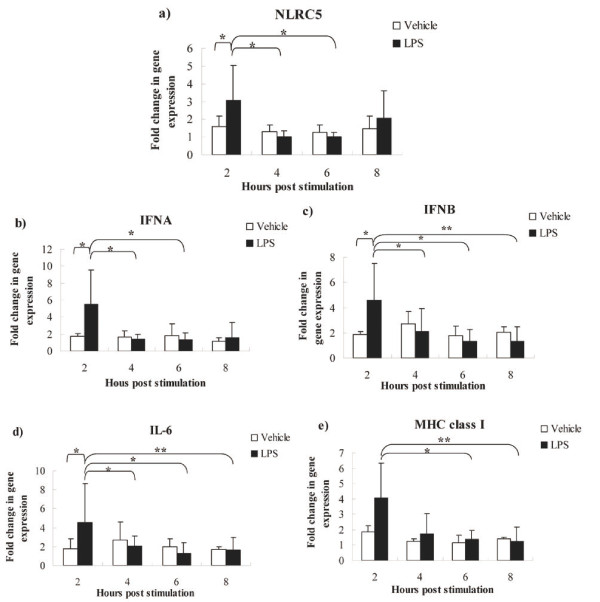
**Gene expression of *NLRC5*, *IFNA*, *IFNB*, *IL-6*, and *MHC class I *after LPS treatment in negative siRNA transfection controls**. HD11 macrophages were stimulated for 2, 4, 6 and 8 hours with 1 μg/ml LPS. Data are shown as the fold change in mRNA expression between LPS treatment and vehicle-treatment for each gene. a: *NLRC5*; b: *IFNA*; c: *IFNB*; d: *IL-6*; e: *MHC class I*; RNA samples were assayed in triplicates by qPCR. P values are flagged with asterisks when lower than 0.05(*) or 0.01 (**).

The expression of *NLRC5*, *IFNA*, *IFNB*, and *IL-6 *was significantly up-regulated, and *MHC class I *showed an increasing trend, in LPS-treated HD11 cells compared to vehicle controls at 2 hours post stimulation (hps) (Figure 1). The expression of *NLRC5 *and *IFNA *after LPS treatment at 2 hps was higher than 4 and 6 hps (Figure 1a,b). *IFNB *and *IL-6 *genes expression at 2 hours post LPS stimulation was higher than 4, 6, and 8 hps (Figure 1c,d). *MHC class I *gene expression was also higher at 2 hours post LPS treatment than these at 6 and 8 hours (Figure 1e). However, poly (I:C) had no effect on gene expression, based upon lack of significant difference between the poly (I:C) treated group and non-poly (I:C)-treated controls at any time points in negative siRNA transfected cells (data not shown), which may be because of miscalculation in the dilution of the stock resulting in the low concentration of poly (I:C) used.

LPS and poly (I:C), as agonists of TLR4 and TLR3, respectively, have been extensively used to mimic bacterial and viral infection to induce inflammation in host innate immune response studies. In mammals, LPS is recognized by LPS-binding protein (LBP)3 and transferred to another LPS binding protein CD14, which delivers the LPS to a complex of myeloid differentiation protein-2 (MD-2) and TLR4 [32]. The TLR4/MD-2/CD14 complex transduces the LPS signal through the recruitment of various adaptor molecules at the Toll/IL-1R (TIR) domain of the TLR4 receptor, and triggers downstream signaling events, such as activation of nuclear factors, NF-kB and AP-1, and subsequent production of proinflammatory cytokines and interferons [33]. Two predominant intracellular pathways, the MyD88-dependent and independent pathways are activated by TLR agonists to induce inflammatory responses [34]. The MyD88-dependent pathway utilizes MyD88 and TIRAP and results in activation of NF-kB and production of the responsive immunoregulatory molecules, such as IL-1, IL-2, IL-6, TNF, and IFNG [35,36]. MyD88-independent pathway, also called the TRAM/TRIF-dependent pathway, induces phosphorylation of IFN regulatory factor (IRF)3 and the subsequent production of type I IFNs as well as a delayed NF-kB response [37-39]. TLR4 is an unique member of TLR family, which activates both the MyD88-dependent and the independent pathways [40,41]. However, TLR3, the sensor of poly (I:C), exclusively triggers the MyD88-independent pathway, leading to production of inflammatory cytokines such as TNF, IL-6, and IFNB [40-42].

Keestra et al. recently reported that a functional LPS-induced MyD88-independent pathway is absent in chicken, based upon their observation of no change of *IFNB *gene expression after LPS treatment (5 μg/ml of *Salmonella Enteritidis *(SE) or *Salmonella Gallinarum *(SG)-LPS) in chicken cells [43]. Interestingly, conflicting results were obtained in the current study, as well as in research recently reported by Esnault et al. [44]. Use of different sources and doses of LPS may partly explain the conflicting results among the studies. In the current study, gene expression of *IFNA*, *IFNB*, and *IL-6 *was up-regulated after *Salmonella typhimurium *(ST)-LPS treatment (1 μg/ml) at 2 hps. These results indicate that LPS stimulation induced elevated expression of type I IFN genes (*IFNA *and *IFNB*) and also an NF-kB-reponsive gene (*IL-6*). Similar results were reported by Esnault et al. [44], who found that the expression of *IFNA*, *IFNB *and *IL-8 *was strongly up-regulated in chicken epithelial cell line (CLEC213) after *E. coli *(EC)-LPS (10 μg/ml) stimulation between 4 hps and 24 hps. Collectively, these studies suggest that specific gene expression patterns after LPS stimulation are dependent upon the distinct LPS sources and dosage.

Additionally, in the present study, LPS treatment up-regulated the expression of the *NLRC5 *gene in chicken HD11 macrophage cells at 2 hps. Although LPS and poly (I:C) are considered to be moderate regulators of *NLRC5*, the reported effects seem to be varied and dependent upon the cell type tested. For example, in mouse splenic B lymphocytes, LPS induced a moderate increase in *NLRC5 *expression compared with IFNG, which is a strong regulator of *NLRC5 *[9]. But in murine bone marrow-derived macrophages (BMDMs), LPS simulation did not alter *NLRC5 *mRNA expression, whereas, poly (I:C) treatment increased its expression [9]. In RAW 264.7 cells, LPS stimulation up-regulated *NLRC5 *at mRNA and protein levels, peaked at 6 hours post treatment, while only a weak increase of *NLRC5 *was observed after poly (I:C) stimulation [27].

### S3-siRNA knocked down *NLRC5 *expression, and *IFNA *and *IFNB *were down-regulated in s3-siRNA transfected cells

**Figure 2:**
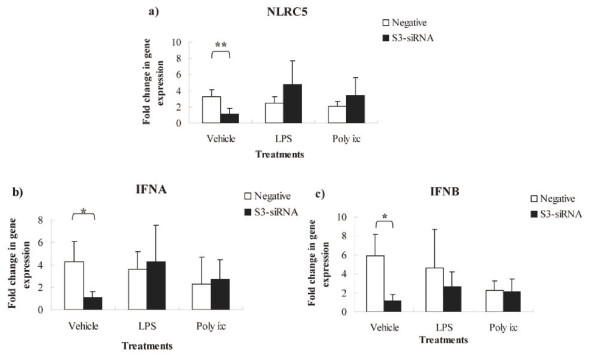
**Gene expression of *NLRC5*, *IFNA*, and *IFNB *in s3-siRNA transfected HD11 macrophages cells after vehicle-treatment, LPS (1 μg/ml), and poly (I:C) (0.1 ng/ml) treatment at 4 hps**. Data are shown as the fold change in expression for each gene between s3-siRNA and negative siRNA transfections, among vehicle-treatment, LPS treatment, and poly (I:C) treatment. a: *NLRC5*; b: *IFNA *c: *IFNB*. RNA assays were carried out in triplicates by qPCR. P values are flagged with asterisks when lower than 0.05 (*), 0.01 (**).

To further investigate the roles of *NLRC5 *in regulating expression of type I IFNs and NF-kB responsive genes in chickens, we utilized RNA interference to knock down *NLRC5 *expression, and then treated cells with LPS or poly (I:C), as well as a vehicle control. The gene expression of type I IFNs (*IFNA *and *IFNB*), an NF-kB responsive gene (*IL-6*), and also an antigen presentation molecule (*MHC class I*) was quantified by qPCR. Of three siRNAs designed, only s3-siRNA down-regulated the expression of *NLRC5 *compared to negative irrelevant siRNA. NLRC5 expression was reduced by 65% at 4 hps (Figure 2a) with a transfection efficiency 75%. Interestingly, *IFNA *and *IFNB *exhibited down-regulated expression patterns consistent with that of *NLRC5 *in s3-siRNA transfected cells (Figure 2b,c). Although gene expression of NLRC5, IFNA, and IFNB in the s3-siRNA LPS treatment group is not significantly higher than that in s3-siRNA vehicle control group (*P *> 0.05), their gene expression has numerically higher values in the s3-siRNA LPS group (Figure 2), and it appeared that the gene expression decrease is relatively alleviated after LPS treatment. These results indicated that knockdown of *NLRC5 *negatively modulated gene expression of *IFNA *and *IFNB *in chicken HD11 cells. There was no significant change in *IL-6 *and *MHC class I *gene expression in *NLRC5 *knocked-down cells.

*NLRC5 *is a highly conserved member of the NLR family and has been reported to be involved in type I IFN and NF-kB signaling pathways, as well as regulating *MHC class I *[9,22-24,26-28]. However, the specific role of *NLRC5 *in regulating inflammatory immune response remains unsolved [24,45]. Cui et al. showed that *NLRC5 *is a negative modulator of NF-kB and the type I IFN pathways. *NLRC5 *potently inhibited the NF-kB pathway through blocking phosphorylation of two subunits of IKK complex, IKKA and IKKB. It also repressed RLR-mediated type I IFN response by interaction with RIG-I and MDA5 [27]. Similar results have been reported that *NLRC5 *overexpression resulted in down-regulation of NF-kB and ISRE in the type I IFN-dependent pathway in HEK293T cells [9]. *NLRC5 *knockdown in RAW264.7 cells induced secretion of proinflammatory cytokines, such as TNF, IL-6, and IL-1B [9]. In contrast, Kuenzel et al. reported that overexpression of *NLRC5 *in epithelial cells induced greater mRNA levels of *IFNA*, *PRKRIR*, and *OAS1 *[25]. siRNA-mediated knockdown of *NLRC5 *significantly impaired induction of *IFNA *in human foreskin fibroblasts cells (HEF) after cytomegalovirus (CMV) infection [25], which indicates *NLRC5 *functions as a positive regulator of type I IFN. The same conclusion was proposed by Neerincx et al. [26], who showed that *NLRC5 *knockdown reduced the secretion of IFNB in THP-1 cells infected with Sendai virus, which predominately induces type I IFNs [46]. However, in the same study, overexpression of *NLRC5 *failed to induce NF-kB, IFNB, IRF3, IRF7, and ISRE in HEK293T cells, which revealed that impact of *NLRC5 *on downstream pathways might depend on the specific cell type examined. Cell-specificity of response was also supported by Kumar et al. [23], who reported that there was no difference in expression of *IFNB*, *IL-6*, and *IFNA *between wild type (WT) and *NLRC5*-deficient mice after infection with RNA viruses, DNA virus, and bacteria in macrophages and dendritic cells. Expression levels of *TNFA*, *IL-6*, and *CCL5 *in GM-CSF-induced bone marrow dendritic cells (GMDCs) after LPS treatment were not different between *NLRC5*-deficient and WT mouse. The authors suggested that *NLRC5 *may be not necessary for induction of inflammatory cytokines under physiologic conditions after viral and bacterial infections [23]. The roles of *NLRC5 *in inflammatory responsive pathways appear to depend on the cell types or specific species [24]. In the present study, expression of *IFNA *and *IFNB *was down-regulated in *NLRC5 *knocked-down HD11 cells. These results suggest that *NLRC5 *positively regulates type I IFNs in the chicken HD11 macrophage cell line. In addition, *NLRC5 *has been shown to regulate *MHC class I *gene expression, but reports on the direction of gene regulation are controversial. *NLRC5 *was reported to positively regulate the expression of *MHC class I *gene through binding to the promoter region of *MHC class I *in lymphoid and epithelial cell lines [28]. However, a conflicting effect was observed in RAW 264.7 cells, where knockdown of *NLRC5 *increased expression of MHC class I on cell surface [9]. In the present study, we detected expression of *MHC class I*, but there was no significant difference between *NLRC5 *knocked-down HD11 cells and controls, regardless of LPS or poly (I:C) treatment. These results indicate that *NLRC5 *is not a major regulator of *MHC class I *in chicken macrophages.

## Conclusion

This is the first study reporting the role of *NLRC5 *in regulating type I IFNs (*IFNA *and *IFNB*), an NF-kB responsive gene (*IL-6*), and antigen presenting pathway gene (*MHC class I*) in the chicken. The expression of both *IFNA *and *IFNB *was down-regulated in *NLRC5 *knocked-down cells, and their expression was relatively restored by LPS treatment (*P *> 0.05). The consistent expression patterns between *NLRC5 *and *IFNA *and *IFNB *indicates that *NLRC5 *is a positive modulator in type I IFN pathway in chicken. In addition, we found that ST-LPS treatment could induce *IFNB *expression in the chicken HD11 macrophage cell line, although a functional LPS-specific MyD88-independent pathway is reportedly absent in chickens. Stimulation with LPS from different sources and doses may be responsible for differing reports on induction of *IFNB *expression.

## Methods

### Cell culture, siRNA transfection, and stimulation of cells with LPS and poly (I:C)

The chicken HD11 macrophage cell line [47], was cultured at 37°C and 5% CO_2 _concentration in RPMI1640 medium (Sigma-Aldrich Co.) supplemented with 10% heat-inactivated fetal calf serum, 10 mM HEPES, 0.1 mM non-essential amino acids, 2 mM glutamine, 1 mM sodium pyruvate, 100 U/ml penicillin, 100 μg/ml streptomycin, and 5 × 10^-5 ^M 2-mercaptoethanol (pH 7.3). Three siRNAs, named s1, s2, s3-siRNA, were designed by using BLOCK-iT™ RNAi Designer (Invitrogen, Carlsbad, CA). s1-siRNA: 5'-CAUGGACGUGUCAUCAGCUUCUAAA-3', s2-siRNA: 5'-GGACGUUUAUCAUGUUGCUAGCUGA-3', s3-siRNA: 5'-CAUAACACUGCAGUCCUGAGGUUUA-3'. Five siRNAs (s1-siRNA, s2-siRNA, s3-siRNA, mixture of s1, s2, s3-siRNAs, and negative-siRNA) at 100 pM were used to transfect cells by following the manufacturer's instructions for use of Lipofectamine™ RNAiMAX (invitrogen). Transfection efficiency was evaluated by using a positive control, BLOCK-iT™ Alexa Fluor^® ^Red Fluorescent after 6 hours of transfection under a fluorescent microscope. Twenty-four hours post transfection, the HD11 cells were exposed to ST-LPS (1 μg/ml) (Sigma-Aldrich Co.) or poly (I:C) (Invivogen, Carlsbad, CA) (0.1 ng/ml), or vehicle as a control. At 2, 4, 6, and 8 hours post simulation, the cells were collected for RNA extraction. Three biological triplicates were used in each group.

### RNA isolation and quantitative reverse transcriptase-PCR

RNA was isolated by using RNAqueous^® ^Kit (Ambion, Austin, TX) followed by DNA treatment using DNA-free™ Kit (Applied Biosystems, Foster City, CA). The expression of *NLRC5*, *IFNA*, *IFNB*, *IL-6*, and *MHC class I *was quantified by qRT-PCR using QuantiTect SYBR Green RT-PCR (Qiagen, Waltham, MA). The primers for *28 s*, *IL-6*, and *MHC class I *have been previously reported [48,49]. The primers specific for the other genes were as follows: *NLRC5 *(F-5'- TGAGCTACACGTCAGGAAGGA-3', R-5'-GCTCTGCAGAATGGACACAA-3'); *IFNA *(F-5'- GACAGCCAACGCCAAAGC-3', R-5'-GTCGCTGCTGTCCAAGCATT-3'); *IFNB *(F-5'- CTGGATTGACCGCACACGCCA-3', R-5'- GGGAGCGCGTGCCTTGGTTTA-3'); Each reaction was run in triplicate and in a final volume of 25 ul with 50 ng/μl or 75 ng/ul total RNA, 12.5 ul QuantiTect SYBR Green master mix, 0.25 ul QuantiTect RT mix, forward and reverse primers, and RNase-free water. Samples were randomly assigned to PCR plates. The slopes for genes were determined with 10-fold serial dilutions. Adjusted cycle threshold (C(t)) values were calculated by following equation: 40 - [C (t) sample mean + (C(t) 28s median -C(t) 28s mean) * (gene slope/28s slope)]. Data were analyzed with the JMP software (SAS Institute, Cary, NC). The main fixed effects were siRNA, treatment, time, and interactions of these effects. Plate was included as a random effect. Multiple comparisons of least squares (LS) means for siRNA, treatment, and time effects were determined by the Tukey-Kramer Honestly Significant Differences test (JMP, SAS Institute, 2005). This test was selected because it allows multiple comparisons among the different treatments (five siRNAs and three treatments including LPS, poly (I:C), and non-treatment controls) without dividing the whole data set, which results in a more robust analysis. Differences were considered to be statistically significant when the *P *value was less than 0.05. Results were described as fold-change determined by 2-ΔΔCt method.

## Abbreviations

AP-1: Activator protein; CCL: Chemokine (C-C motif) ligand; GM-CSF: Granulocyte-macrophage colony-stimulating-factor; IKK: Inhibitor of kappa B (IkB) kinase complex; MDA: Melanoma differentiation-associated protein; MHC: Major histocompatibility complex; MyD88: myeloid differentiation primary response gene 88; NF-kB: Nuclear factor kappa-light-chain-enhancer of activated B cells; OAS1: 2'-5'-oligoadenylate synthetase 1; PRKRIR: protein-kinase, interferon-inducible double stranded RNA dependent inhibitor, repressor P58 repressor; TIRAP: TIR domain containing adaptor protein; TRAM: TRIF-related adapter molecule; TRIF: TIR-domain-containing adapter-inducing IFNB.

## Authors' contributions

LL carried out the experiments and data analysis for qPCR, and drafted the manuscript. CC designed the study and participated in the experiment, data interpretation, and revision of the manuscript. GBC performed a pre-experiment. JDH participated in qPCR experiments. SJL participated in the design of the study, data interpretation and revision of the manuscript. All authors read and approved the final manuscript.
